# Skin autofluorescence assessment of cardiovascular risk in people with severe mental illness

**DOI:** 10.1192/bjo.2018.34

**Published:** 2018-07-25

**Authors:** Daniëlle Emmerink, Sybiel Bakker, Thomas Van Bemmel, Eric O. Noorthoorn, Paul Naarding

**Affiliations:** Psychiatrist, Department of Old-Age Psychiatry, GGNet Mental Health, Apeldoorn, The Netherlands; Nurse Practitioner, Department of Internal Medicine, Gelre Hospital, Apeldoorn, The Netherlands; Internist, Department of Internal Medicine, Gelre Hospital, Apeldoorn, The Netherlands; Senior Researcher, Department of Old-Age Psychiatry, GGNet Mental Health, Apeldoorn, The Netherlands; Psychiatrist, Department of Old-Age Psychiatry, GGNet Mental Health, Apeldoorn, The Netherlands and Department of Psychiatry, University Medical Centre Groningen, The Netherlands

## Abstract

**Background:**

People with severe mental illness (SMI) show significantly shorter life expectancy, mostly due to more prevalent cardiovascular disease. Although age is a prominent contributor to contemporary risk assessment and SMI usually affects younger people, these assessments still do not reveal the actual risk. By assessing advanced glycation end products (AGEs), cardiovascular risk can be assessed independent of age.

**Aims:**

To establish whether detection of AGEs with the AGE-reader will give a more accurate cardiovascular risk assessment in people with SMI.

**Method:**

We compared assessment with the AGE-reader with that of the Systematic Coronary Risk Evaluation (SCORE) table in a group of 120 patients with SMI.

**Results:**

The AGE-reader showed an increased cardiovascular risk more often than the SCORE table, especially in the youngest group.

**Conclusions:**

Because of its ease of use and substantiation by studies done on other chronic diseases, we advocate use of the AGE-reader in daily care for patients with SMI to detect cardiovascular risk as early as possible. However, the findings of the current study should be evaluated with caution and should be seen as preliminary findings that require confirmation by a prospective longitudinal cohort study with a substantial follow-up observation period.

**Declaration of interest:**

None.

Severe or serious mental illnesses (SMI) are those mental illnesses that show significant mental signs and symptoms for a long(er) period and that lead to significant functional limitations and disability. The prevalence of SMI in the Dutch population is about 1%. Most people with an SMI have schizophrenia or other psychotic disorders, mood (including bipolar) disorders or personality disorders.^1^ The life expectancy of people with SMI is estimated to be 13–30 years shorter compared with the general population.^2^ About 60% of the total deaths among people with SMI are due to natural causes, with cardiovascular disease the most frequent cause of premature death.^3^ The INTERHEART study showed that abnormal lipids, smoking, hypertension, diabetes, abdominal obesity, the presence of psychosocial factors, the lack of consumption of fruit and vegetables, alcohol consumption and the lack of regular physical activity account for more than 90% of the population with attributable risk of an initial acute myocardial infarction.^4^ The incidence of all of these risk factors is higher in people with SMI.

Early detection and treatment of these risk factors can therefore result in significant health benefits, especially in people with a high primary cardiovascular risk, for example those with SMI. In the Netherlands, as in many high-income countries, guidelines are available for general practitioners (GPs) to identify people at high risk: the Dutch Nederlands Huisartsen Instituut (NHG) Guideline Cardiovascular Risk Management (CVRM). The risk profile is primarily based on an assessment of traditional risk factors such as blood pressure, lipids, smoking habits, blood glucose levels and a family history of cardiovascular disease. The internationally accepted Systematic Coronary Risk Evaluation (SCORE) table is part of this guideline. It indicates the 10-year risk of death from cardiovascular disease and serves as a tool for GPs to determine whether or not treatment is necessary.^5^

An alternative method for assessing cardiovascular risk is to measure the amount of advanced glycation end products (AGEs) in the skin. Formation of AGEs occurs in all tissues and body fluids and is part of healthy aging, but they also play a pivotal role in the development of chronic age-related diseases such as diabetes, renal failure and cardiovascular disease.^6^ Many AGEs have characteristic tissue autofluorescence. The AGE-reader (DiagnOptics BV, Groningen, the Netherlands) is able to measure this tissue fluorescence in a fast, easy and non-invasive way.^7^

Several clinical studies demonstrate that skin tissue autofluorescence is a strong and independent risk predictor for mortality, diabetic complications and cardiovascular events.^8^^–^^12^ Some earlier studies have investigated skin tissue autofluorescence in people with psychiatric disorders; those with schizophrenia were found to have higher levels of AGEs, and it was suggested that the increased level of oxidative stress in this group gives rise to these higher levels.^13^^,^^14^ Higher levels of skin tissue autofluorescence have also been associated with depressive disorders; higher levels of AGEs were independently associated with the severity of depression as well as with diagnosis of depression in a group of older people with depression.^15^ In both studies, emphasis was placed on the aetiological role of AGEs in the development of psychopathology. We are interested in the specific somatic needs of people with SMI, especially with respect to cardiovascular risk. Because people with SMI frequently experience their illness and its related behaviour at a younger age, and since cardiovascular risk estimation by SCORE is largely driven by age, we assume that the real cardiovascular risk has been underestimated. We hypothesised that the cardiovascular risk of patients with SMI can be estimated more accurately by using the AGE-reader.

## Methods

### Participants

Participants were all in-patients in long-stay wards of GGNet, a mental health institute in the Netherlands. They were recruited between April and June 2015, and all were 18 years or older and met the criteria as stated in the Dutch consensus on the definition of patients with an SMI.^1^. This means that there is a long-lasting and persisting psychiatric disorder with persistent symptoms and signs, with a considerable impact on daily functioning (Global Assessment of Functioning score of less than 70) and with an enduring need for professional help. Eligible participants were informed about this research through an information letter and permission was requested for the principal investigator to approach them. If they agreed, the principal investigator approached the participant, checked for exclusion criteria and asked for informed consent. Exclusion criteria were a history of cardiovascular disease, diabetes and renal failure (serum creatinine >180 or estimated glomerular filtration rate of <40 mL/min) because, by definition, these characteristics would lead to a high cardiovascular risk and the SCORE would not be suitable. This study was limited to participants with white skin because dark skin has been known to influence skin tissue autofluorescence assessment due to light absorption.^6^ People with a large number of skin defects on their forearms were also excluded because this complicates skin autofluorescence. Finally, patients were excluded if they were unable or unwilling to provide their written consent. Medical ethical approval for the study was provided in 2015 by the ethical board of the University of Twente, Enschede, the Netherlands.

### Procedures

#### Cardiovascular risk assessment with the SCORE table

The Dutch NHG Guideline CVRM is an evidence-based guideline from the Dutch College of General Practitioners dated January 2012, and it is currently the gold standard for GPs to determine increased risk of cardiovascular disease. Within this guideline, the SCORE table combines data on age, gender, systolic blood pressure, smoking status and total cholesterol/high-density lipoprotein (HDL) ratio to estimate the 10-year risk of a severe cardiac outcome.^5^ Using the SCORE table, participants can be divided into three categories, namely low (<10%) intermediate (10–20%) or high risk (≥20%), based on cardiovascular morbidity or mortality in the coming 10 years. All in-patients with SMI in our institute undergo an annual somatic screening in which the above-mentioned cardiovascular risk factors are measured. The data from the last annual somatic screening of all participants were used to determine the cardiovascular risk with the SCORE table and to classify them in one of the three risk groups.

#### Cardiovascular risk assessment with the AGE-reader

The AGE-reader is a desktop box that illuminates 4 cm^2^ of the skin's surface on the forearm while protecting against surrounding light with an excitation light in the 300–420 nm range. This light activates the fluorescent constituents of certain AGEs in tissue, which then begin emitting light of a different wavelength. The emitted light, with an intensity between 420–600 nm, is detected using a spectrometer. Skin tissue autofluorescence is then calculated as the ratio between the total emission intensity and the total excitation intensity multiplied by 100. Through the use of calibrated spectrometers, the results obtained by different AGE-reader standard units are similar. When the skin reflectance R is <12%, an additional reflection measurement is carried out with a white light-emitting diode to provide a more reliable skin tissue autofluorescence value. Each AGE-reader standard unit has a unique calibration file that is stored on the internal flash drive. Reference values are based on 456 measurements in Caucasian participants without cardiovascular disease (smokers and non-smokers).^16^

Three repeated measurements were performed on an area of pale, healthy skin on the forearm, with the participants in the sitting position. The mean of three measurements was used for further analysis. The AGE-reader reveals four categories: normal risk, slightly increased risk, increased risk or high risk.

#### Statistical analyses

We conducted a comparative cohort study on the usability, sensitivity and specificity of the AGE-reader in participants with SMI. Cardiovascular risk assessed with the SCORE table was compared with that of that of the AGE-reader.

Because the CVRM identifies three categories (high, medium and low) and the AGE-reader provides four categories (normal, slightly increased, increased and high risk), we combined the slightly increased and increased scores from the AGE-reader into one category to allow for comparison. Analysis centred on the degree of similarity of the categories in percentages, but also included the degree of similarity corrected for prevalence by using the intraclass correlation. We also conducted a nonparametric Wilcoxon *U*-test (dis-)agreement. We compared the basic data, but also added 15 years to the age categories in the CVRM score tables, following studies in diabetes and rheumatoid arthritis. Furthermore, we compared risk using the transformed (+15 years) CVRM SCORE tables in the sample of participants over the age of 40. Finally, to investigate the association of age to the difference between CVRM and AGE-reader scores, we performed a linear regression on age using difference as the outcome.

## Results

### General characteristics of the study group

We selected 310 participants with SMI in the long-stay wards of our institute. Of these, 93 refused to participate and 74 were excluded because of exclusion criteria. Finally, there was no information on vascular risk factors from a recent annual screening for 23 of the participants, so the final study group consisted of 120 participants. Table 1 shows the characteristics of the study participants. As expected, most participants suffered from F2 psychotic disorders according to ICD-10 (1992). Many showed F1 psychoactive drug use and a large number of participants showed F6 personality disorders or F7 mental handicap. F3 mood disorders and F0 organic mental disorders occurred infrequently. The majority of the participants were male, smokers and over 40 years of age (mean age 47.8, s.d. 15.7). Scores on various components of the SCORE table (blood pressure, total cholesterol/HDL ratio and smoking habits) are given along with the subsequent risk category on the SCORE table. The risk score obtained with the AGE-reader is given next to this in Table 1.

**Table 1:** Study participants

Item	Mean, s.d. or percentage
Number of participants	120
Age (mean – s.d.)	47.8 (15.7)
Age (% over 40 years)	70.8%
Gender (% male)	63.7%
Smoking	65%
Diagnoses ICD-10	
F0: Organic mental disorder	6.7%
F1: Mental disorder due to psychoactive substance abuse	30%
F2: Psychotic disorder	66.2%
F3: Mood disorders	15%
F6: Personality disorders	18.3%
F7: Mental handicap	18.3%
F8: Disorders of psychological development	19.2%
F99: Unspecified mental disorder	8.3%
Number of disorders	1.8 (0.9)
Respiratory rate systolic (mean – s.d.)	130.2 (14.7)
Respiratory rate diastolic (mean – s.d.)	80.4 (8.9)
Ratio high-density lipoprotein to cholesterol (mean – s.d.)	4.9 (1.9)
CVRM risk assessment	
<10% risk	73.3%
10–20% risk	12.5%
>20% risk	14.2%
AGE-reader risk assessment	
Normal risk	28.3%
Slightly higher risk	26.7%
Higher risk category	29.2%
Highest risk category	15.8%

### Comparison of risk assessment with the SCORE table from the NHG Guideline CVRM *v.* the AGE-reader

Table 2 describes the degree of agreement between the two risk assessments in several subpopulations. The table shows that the percentage of similarity between the risk assessment with the SCORE table and the AGE-reader is 27%. In 60% of participants, the risk estimated with the AGE-reader is higher than with the SCORE. Even if an additional 15 years of age are added to the SCORE table, as is done for chronic diseases such as diabetes and rheumatoid arthritis, the AGE-reader still estimates a higher risk in 45% of participants. If this comparison is done in people over 40 only, the AGE-reader estimated a higher risk in 30% of participants. In line with this, the difference in results between the AGE-reader and the SCORE table with increasing age was significantly reduced: β = −0.534, *P* < 0.0001. The time taken for measurement with the AGE-reader was significantly less than that for the SCORE table (*t* = −14.7, d.f. = 119, *P* < 0.001) (data not shown).

**Table 2:** Agreement (%) and intraclass correlation between the AGE-reader and the CVRM analysed on source data and correcting by 15 years

			AGE-reader finding			Totals
			Normal	Increased	High	
CVRM finding	Source data	<10% risk	19 (15.8%)	61 (50.8%)	8 (6.7%)	88
		10–20% risk	7 (5.8%)	5 (4.2%)	3 (2.5%)	15
		>20% risk	8 (6.7%)	1 (0.8%)	8 (6.7%)	17
		Totals	34	67	19	120
	+15 years	<10% risk	11 (9.2%)	37 (30.8%)	3 (2.5%)	51
		10–20% risk	14 (11.7%)	15 (12.5%)	14 (11.7%)	43
		>20% risk	9 (7.5%)	15 (12.5%)	2 (1.7%)	26
		Totals	34	67	19	120
	+15 years in >40 sample	<10% risk	4 (4.7%)	12 (14.1%)	2 (2.4%)	18
		10–20% risk	14 (16.5%)	15 (17.6%)	14 (16.5%)	43
		>20% risk	9 (10.6%)	13 (15.3%)	2 (2.4%)	24
		Totals	27	40	18	85

## Discussion

In this study, we found that the AGE-reader identifies an increased risk of cardiovascular risk in participants with SMI more often than the SCORE table. This is even more notable in the younger group of SMI participants. We conclude that the traditional risk assessment may not be adequate for the group of SMI patients, especially those in the younger age groups. The AGE-reader has been substantially validated in clinical studies, and this preliminary report advocates the use of the AGE-reader in clinical practice for the disease management of people with SMI. However, a longitudinal prospective cohort study is of course necessary to substantiate a predictive value of the AGE-reader in this group.

Accumulation of AGEs is linked to the pathophysiology of a variety of diseases including diabetes, renal disease, cardiovascular diseases and neurodegenerative disorders.^8^^,^^17^^–^^19^ The AGE-reader has been validated against the gold standard for measuring AGEs (skin biopsies). Skin tissue autofluorescence was shown to be strongly correlated with tissue accumulation of AGEs.^6^^,^^9^ More recently, awareness of the possibility of the aetiological role of AGEs in various kinds of psychopathology has increased. The formation of AGEs is linked to increased oxidative stress. This oxidative stress can be the result from several factors, including repeated hyperglycaemia, smoking and hypertension, but probably some psychosis-related factors as well.^12^^,^^14^ Smoking and other unhealthy lifestyle habits may be rather extreme in the subgroup of institutionalised patients with SMI, thus leading to excessive formation of AGEs.^20^

### Clinical implications of the study

For everyday clinical practice, the early detection of the cardiovascular risks of institutionalised patients with SMI is of major importance. Many of these patients may be unable to care for themselves and/or may have an unhealthy lifestyle. Any specialised centre targeting patients with SMI should therefore include an integrated, somatic program. This program should include prevention (targeted at lifestyle, physical exercise and education), early detection (with regular routine controls of cardiovascular parameters, e.g. with the AGE-reader) and treatment (of cardiovascular risk factors, e.g. hypertension, diabetes and hypercholesterolemia). Early detection of risk is crucial because the consequences of all of these risk factors are almost always suffered many years later.

### Strengths and limitations

The strength of our study is that we are among the first to analyse AGEs in a large group of people with SMI and were able to compare the results with a traditional method of cardiovascular risk assessment. We have demonstrated that risk assessment with the AGE-reader is feasible and possible in a group of institutionalised people with SMI.

There are several limitations of this study that need to be addressed: first, a large number of the patients in our study refused to participate. They did not differ in age, gender or diagnosis, but did possibly differ in regard to disease severity and psychosis-related hostility. This could justify the suggestion that we overlooked a group of people that is even more at risk. In addition to this, the AGE-reader is not suitable for dark skin; we had to exclude several people because of this exclusion criterion. This will generate some problems in clinical practice, especially in regions in which more people with SMI have dark skin.

### Conclusion

The primary reason for the underestimation of cardiovascular risk in people with SMI is due to the fact that age is a major contributor to this estimation, and many people with SMI are below the age of 50. Nevertheless, life expectancy is much lower in people with SMI, and cardiovascular disease is the most frequent cause of premature death in people with SMI. This study shows that the AGE-reader identifies an increased risk more often than the SCORE table, especially in younger people with SMI. Despite evidence of the predictive value of the AGE-reader investigated in people with no mental disorders, we may expect the findings to have some validity in the population with SMI investigated in this study. We must keep in mind that these are merely preliminary findings that require confirmation in a prospective cohort study with sufficient follow-up data. However, using the AGE-reader to assess cardiovascular risk in people with SMI at a younger age could improve early recognition of such risks, therefore we would advocate using this method of risk assessment. An additional benefit is that the AGE-reader allows a quick and non-invasive assessment of the AGE accumulation in skin in less than a minute.
